# Cancer: Linking Powerhouses to Suicidal Bags

**DOI:** 10.3389/fonc.2017.00204

**Published:** 2017-09-06

**Authors:** Laia Martinez-Carreres, Anita Nasrallah, Lluis Fajas

**Affiliations:** ^1^Cancer and Metabolism Laboratory, Center for Integrative Genomics, University of Lausanne, Lausanne, Switzerland

**Keywords:** cancer, mitochondria, lysosomes, mammalian target of rapamycin, Rab7, AMP-activated protein kinase, mitophagy, lysosomal membrane permeabilization

## Abstract

Membrane-bound organelles are integrated into cellular networks and work together for a common goal: regulating cell metabolism, cell signaling pathways, cell fate, cellular maintenance, and pathogen defense. Many of these interactions are well established, but little is known about the interplay between mitochondria and lysosomes, and their deregulation in cancer. The present review focuses on the common signaling pathways of both organelles, as well as the processes in which they both physically interact, their changes under pathological conditions, and the impact on targeting those organelles for treating cancer.

## Introduction

Cancer is characterized by the unrestricted cellular growth and proliferation of abnormal cells. It exhibits properties of motility, invasion, angiogenesis, and metastasis. Recent studies identified diverse mechanisms of metabolic plasticity in cancer cells. These include increased glucose uptake in most tumors, elevated glycolytic intermediates, increased pentose phosphate pathway activities, increased glutamine catabolism, and increased use of lactate as a fuel in selective tumors (1).

According to the American Cancer Society, it is estimated that, in the US, almost 1.7 million new cases of cancer will be diagnosed in 2017. Mostly, general cancer treatments are limited to radiation, chemotherapy, and surgery. However, these treatments encounter non-specific distribution of chemotherapeutic agents, insufficient drug concentrations to reach the tumor, and restricted ability to survey therapeutic responses (2). More efforts are targeted to find new therapies to help overpass these obstacles. Subcellular targeting is beneficial for therapy in several scenarios (3): (1) basic organelle malfunctions could be targeted, making the process more selective; (2) the quantity of drug required could be significantly reduced because of its specificity, which eventually helps in decreasing side effects; and (3) most importantly, intracellular drug targeting may surpass dangerous drawbacks of drug actions in cancer therapy, i.e., multidrug resistance (4, 5).

In most of the mechanisms of cancer initiation and progression, different organelles are involved, especially mitochondria and lysosomes, for their relevance in energy homeostasis and cell death (6). The purpose of this review is to shed light on the roles of mitochondria and lysosomes in cancer, as well as them being prominent targets for cancer therapy.

## The Role of Mitochondria in Cancer

Mitochondria, also called “powerhouses” of cells, are double membrane organelles, with their own genome, thought to have been originated from an ancient symbiosis that resulted when a nucleated cell engulfed an aerobic prokaryote. Through evolution, mitochondria conserved only a small part of prokaryotic bacterial genes, including the ones encoding 13 proteins of the respiratory chain (7). In this manner, mitochondria gained a central role in the regulation of metabolism, cell proliferation, and apoptosis, while many tasks were transmitted to the host cells (8).

Other than being cell’s powerhouses, mitochondria function as signaling organelles. They coordinate distinct metabolic pathways, producing metabolites required for cell survival and proliferation (9). In fact, mitochondria are key players in the calcium-signaling pathway (10). When toxic stimuli damage the cell, mitochondria release pro-apoptotic molecules, such as cytochrome *c*, thus regulating cell death (11). Moreover, mitochondria are established as the major site of production of free radicals, which are major signaling molecules in the cell (12). Recently, it has been shown that mitochondrial metabolites do not only have intermediary roles in energy generation but can also promote regulatory effects on post-translational modifications of proteins (13), as well as affecting chromatin structure and function (14).

Multiple human diseases have been strongly associated with impaired mitochondrial homeostasis. These include liver and cardiovascular diseases, neurological and muscular disorders, seizures, susceptibility to infections, and cancer (15–18). In cancer, mitochondrial roles vary as a function of genetic and environmental differences, as well as the tissue-of-origin of the diverse types of cancer. The main mitochondrial processes contributing to tumorigenesis include mitochondrial biogenesis and mitophagy, fission and fusion dynamics, metabolism, oxidative stress, and cell death (19).

Compared to normal cells, cancer cells show many alterations in energy metabolism. In the 1920s, cancer metabolism studies commenced with Otto Warburg’s observation: to produce energy, cancer cells rely less on mitochondrial respiration and more on glycolysis. Warburg hypothesized that mitochondria must be dysfunctional, taking into consideration that glycolysis gives a lot less energy as compared to mitochondrial respiration (20). However, other scientists believed that the reduced mitochondrial activity is due to higher glycolysis. In some cases, Warburg’s proposal holds true. Nevertheless, there are reports showing that the mitochondrial function in cancer cells in some cases is intact, or mitochondrial biogenesis is increased (21).

Mitochondrial biogenesis could be described as the division and growth of pre-existing mitochondria. It is regulated at the transcriptional and post-transcriptional levels of gene expression (22). Regulating mitochondrial biogenesis is an attractive target of key oncogenic signaling pathways, since cancer cells induce it to increase ATP production for cellular proliferation. PGC-1α, through its interactions with numerous transcription factors, is a central regulator of mitochondrial biogenesis (23). It portrays a dual effect on cancer viability. On the one hand, PGC-1α acts as a tumor suppressor in some cancers, resulting in induced apoptosis upon overexpression. In human epithelial ovarian cancer, apoptosis was induced *via* the organized regulation of Bcl-2 and Bcl-2-associated X protein (BAX) expression by PGC-1α (24). PGC-1α is considered a tumor suppressor not only because it induces apoptosis but also because it has been found to suppress the metastatic abilities of tumor cells *via* the direct regulation of transcriptional machinery (25, 26). For example, PGC-1α directly increases ID2 transcription that binds to the transcription factor TCF4, rendering it inactive. This in turn leads to a downregulation in metastasis-related genes, such as integrins, that are able to influence metastasis and invasion (25). On the other hand, the ability of PGC-1α in sustaining metabolic homeostasis can also promote cancer cell survival and tumor metastasis (27). In cancer cells, silencing PGC-1α resulted in deferred invasive potential and weakened metastatic ability without affecting proliferation and tumor growth. Consistently, the transition from primary lung tumor cells to metastatic cancer cells was coupled with more dependence on mitochondrial respiration, *via* PGC-1α, leading to an upregulation of PGC-1β, ERRα, and NRF1, which are mitochondrial-related biogenesis genes (28).

Another key activator of mitochondrial biogenesis in cancer is c-Myc, a transcription factor regulating cell cycle, proliferation, metabolism and cell death. Studies have demonstrated that the loss or gain of Myc decreases or increases mitochondrial mass, respectively. This is due to the fact that over 400 mitochondrial genes are identified as targets of c-Myc (29). A third effector of mitochondrial biogenesis is mammalian target of rapamycin (mTOR). It controls mitochondrial gene expression through the activation of PGC-1α/YY1 and represses the inhibitory 4E-BPs (eukaryotic translation initiation factor 4E-binding protein 1) that downregulates the translation of mitochondrial proteins (30).

During tumorigenesis, mitochondrial dynamics is very important. It determines the equilibrium between cell death programs and mitochondrial energy production. Several studies demonstrated, in cancer, an imbalance in mitochondrial fission and fusion activities, depicted in decreased fusion, and/or elevated fission that resulted in fragmented mitochondrial networks *via* the K-Ras-DRK1/2-Drp1 pathway (31, 32). Also, c-Myc affects mitochondrial dynamics by altering the expression of proteins implicated in the fission and fusion processes (33).

Furthermore, mitochondria have a tight relationship with the intrinsic (also called mitochondrial) apoptotic cell death program, since B-cell lymphoma-2 (BCL-2) family of proteins regulates the integrity of the outer mitochondrial membrane (OMM). Mainly two members of this family, BAX and Bcl-2-associated killer (BAK) can break the OMM in response to apoptotic stimuli. This releases apoptogenic factors from inside mitochondria, such as cytocrome *c*, inducing activation of caspases and subsequent cell death. In some cases, mitochondria can also participate in the extrinsic apoptotic pathway, which is initiated by cell membrane death receptors. For example, FAS receptor can truncate Bid protein, another member of the BCL-2 family, *via* caspase 8. Truncated Bid (tBid) can then translocate to mitochondria to induce apoptosis (34).

Mitochondrial morphology is a hallmark for apoptotic susceptibility. Even though fission and fusion do not regulate apoptosis *per se*, the generated mitochondrial morphology supports the interaction with pro-apoptotic Bcl-2 proteins. Thus, mitochondrial hyper-fragmentation causes resistance to apoptosis due to the inability of mitochondrial membranes to interact with pro-apoptotic proteins (35).

Mitochondria play major roles in metabolic reprograming, including the synthesis of macromolecules and cellular survival (1). One mechanism by which cancer drives these alterations in metabolism is limiting pyruvate utilization by the mitochondria. This is achieved by regulating pyruvate kinases such as PKM isoforms (36), as well as downregulating mitochondrial pyruvate carriers: MPC1 and MPC2 (37). Moreover, in some tumor types, mutations in the enzymes of the tricarboxylic acid (TCA) cycle render the mitochondria dysfunctional. Cells from such tumors use glutamine-dependent reductive carboxylation rather than oxidative metabolism as the major pathway of citrate formation. This, in turn, leads to the major reprograming of amino acid metabolism and lipid synthesis (38).

There are multiple levels at which both mitochondrial biology and tumorigenic signaling vastly intersect. First, cellular physiology and tumorigenesis are affected by direct signals from mitochondria. Metabolites generated by the mitochondrial pathways affect gene transcription through chromatin modification, and cytosolic signaling pathways (19). For example, the TCA cycle intermediate α-ketoglutarate (α-KG) is a co-substrate for many enzymes in the cytoplasm and nucleus, including families of chromatin-modifying ones. In the case of chromatin regulation, glutamine-derived α-KG contributes to TET-dependent demethylation reactions (38). Second, many mutations were identified as directly associated with cancer risk (39). Cancer can be caused by mutations in nuclear-encoding genes, such as electron transporter chain (ETC) genes. For example, patients suffering from paraganglioma often presents dysfunctions in succinate dehydrogenase (SDH). Mutations of the same complex have also been found in other cancers, such as gastrointestinal stromal cancer, breast cancer, or renal carcinomas. Other enzymes such as fumarate hydratase (FH) have also found to be mutated in other cancers. When the function of these enzymes is lost, the metabolic intermediates fumarate and succinate accumulate, which in turn function as oncometabolites when found in excess (40, 41). In addition, mitochondrial DNA mutations (amplifications, deletions, point mutations, etc.) have been associated with various cancers (42). For example, point mutations in MT-ND1 gene modify complex I activity, having an influence on the tumorigenic characteristics of cells. Finally, and in order to support tumorigenesis, classical oncogenic signaling pathways alter mitochondrial functions. These include the c-Myc, p53, mTOR, and k-Ras signaling pathways (19). In addition, a main function of mitochondria is synthesizing aspartate for nucleotide synthesis, inducing cellular proliferation (43).

Mitochondria are complex organelles affecting cancer at many levels: initiation, proliferation, survival, or metastasis. One type of the various organelles that communicate with mitochondria is lysosomes. Mainly, this crosstalk depends on mitochondrial stress and/or destabilization of lysosomal membranes (44).

## The Role of Lysosomes in Cancer

Also known as “suicidal bags,” lysosomes were first described in 1950s by Christian de Duve as membrane-enclosed vesicles containing hydrolases. Functioning as a digestive system, they are found in all eukaryotic cells, except for mature erythrocytes. The hydrolytic enzymes that they contain include proteases, nucleases, and lipases that can break down proteins, nucleic acids, and lipids, respectively, to their simplest subunits (45).

Lysosomes are formed when material from outside the cell is internalized in clathrin-coated endocytic vesicles forming early endosomes. Endosomal maturation occurs with the delivery of lysosomal acid hydrolases from the trans Golgi network, which contribute lowering of the internal pH to about 5.5. Late endosomes then mature into lysosomes as they acquire a full complement of acid hydrolases, which digest the molecules originally taken up by endocytosis, phagocytosis, and autophagy (46). Nevertheless, many investigations have proved that lysosomes are not only degradative organelles but also participate in metabolism of the entire cell at different levels, and their modifications can promote or repress cell proliferation.

On one hand, lysosomes undergo Ca^2+^ regulated exocytosis, which is secreting their content into the extracellular space, and repairing their damaged plasma membranes; when the plasma membrane is injured, lysosomes quickly move to the site of damage and fuse with the plasma membrane. This allows effective resealing (47). On the other hand, they can sense nutrient availability, which controls energy metabolism and mediates the starvation response (48). Zoncu et al. proposed that amino acids have to be detected in the lysosomal lumen, signaling to the Rag GTPases in a manner that is vacuolar H^+^-ATPase (V-ATPase)-dependent. This is known as the “inside-out” mechanism (49). Leucine, among other amino acids, must accumulate in the lumen of the lysosome to trigger the central regulator of cellular and organismal growth, mammalian target of rapamycin complex I (mTORC1) (50). mTORC1 is recruited by Rag GTPases on the lysosomal surface in response to amino acids, the site of activation by Rheb (Ras homolog enriched in brain), when growth factor-stimulated PI3K–Akt signaling is on (51, 52). Upon amino acid and growth factors removal, Rag GTPases releases mTORC1, causing it to become cytoplasmic and inactive. In those conditions, the negative regulator of Rheb, tuberous sclerosis complex 2 (TSC2), is lysosomally localized. Thus, lysosomal proteins change depending on the nutrient status of the cells (53, 54).

Lysosomal biogenesis as well as autophagy is controlled by the main regulator of lysosomal genes, known as TFEB or transcription factor EB; when mTORC1 is active, TFEB remains inactive at the lysosomal membrane. Inactivated mTORC1 induces TFEB localization to the nucleus to activate lysosomal gene transcription (52, 55).

Since lysosomes also serve as platforms of activation of mTORC1, it is important to mention the dysregulation of this pathway in cancer. Indeed, mTORC1 regulates several anabolic processes that are critical for tumorigenesis: it promotes protein synthesis, aerobic glycolysis, *de novo* lipid synthesis, *de novo* nucleotide synthesis, and represses autophagy and lysosomal biogenesis (56–59). Genes that encode components of the PI3K–Akt–mTOR pathway are frequently mutated in cancer, but despite few mutations have been characterized in mTOR, many tumor types present mTOR hyperactivation, thus promoting tumorigenesis (60, 61).

In addition, lysosomal intracellular positioning is important for adhesion and motility (62), and important for mTOR signaling, autophagosome formation, and autophagosome-lysosome fusion, and changes depending on the nutrient availability. During starvation, mTORC1 activity is repressed, which induces autophagosome formation. Starvation increases pH, causing lysosomes to cluster near the microtubule-organizing center (MTOC), facilitating autophagosome–lysosome fusion. Conversely, nutrient replenishment restores basal pH inducing lysosomal scattering, which brings lysosomal mTORC1 to the cell periphery and stimulates its activity by increasing its coupling to the gradient of signaling molecules emanating from the plasma membrane (63). Given that peripheral lysosomes inside the cell are responsible for cell adhesion and motility, targeting those lysosomes in cancer cells is also a good strategy for cancer treatment (62).

As de Duve already stated in the 1950s, lysosomal membrane permeabilization (LMP), consequently leading to the leakage of lysosomal content into the cytoplasm, induced what is known as “lysosomal cell death” (45, 64). Major players of this mechanism are lysosomal cathepsin proteases. They have apoptotic and/or necrotic features, depending on the cellular context and the extent of leakage occurring into the cytosol (65).

Lysosomes in cancer cells undergo major changes. In some cases, they have an increased volume and protease activity, along with an improved lysosomal protease secretion, as compared to lysosomes in normal cells. Thus, they become hyperactivated as a reaction to fulfill the needs of the challenging microenvironment of the tumorigenic cells (62). For example, they require the ingestion of huge amounts of adhesion molecules and extracellular matrix molecules, leading to an upregulation in exocytosis. Also, they have to move inside the cell to repair damaged membranes (66, 67). Recently, a correlation between lysosomal movement and tumor cell invasion was also established, which was induced by tumor microenvironment stimuli (68). In particular, acidic extracellular pH induced lysosomal movement toward the cell peripheries, successively leading to Cathepsin B exocytosis from the lysosomes. This eventually promoted protease-dependent tumor invasion (69, 70). *In vitro* studies with glioma cells have shown that inhibition of lysosomal exocytosis with vacuolin-1 is a good strategy for fighting against invasion in cancer (71).

As explained above, changes in the lysosomal compartment, in the presence of increased secretions of cysteine cathepsins, render these lysosomes pro-oncogenic. This results in an increased neoplastic progression, *via* proteolytic pathway initiation (72). Other than matrix remodeling, lysosomal role in degradation is crucial in tumorigenesis. This has been observed in reports revealing that specific intracellular cathepsin inhibitors are able to block collagen degradation, promoting tumor viability (73). However, cathepsins are also depicted as proteases with tumor suppressor abilities for their role in inducing cell death through LMP (74, 75).

Due to their role in cell death, autophagy, and deregulating metabolism, targeting lysosomes have a great therapeutic potential in cancer. Lysosomal proteins are indeed good targets for cancer treatment (76), such as lysosome-associated membrane protein 1 (LAMP-1). LAMP-1 is suggested to have a role in cell–cell adhesion and migration, since it was detected on the surface of highly metastatic cancer cells, particularly from colon cancer (77). V-ATPase is another significant lysosomal membrane protein participating in cancer. It functions as a pump of protons to create an acidic pH of lysosomes. Also, it regulates endocytotic trafficking and affects the tumor microenvironment, by extruding protons into the extracellular matrix (78). Moreover, in tumor malignancy, V-ATPase participates not only in the dysregulation of lysosomal trafficking but also in mTORC1 activation and autophagy (79).

In addition, lysosomes are key players in cancer drug resistance. They can sequester cancer drugs into their acidic milieu, thus, blunting the drugs’ effects (80). This further proves that targeting lysosomes may be a promising new therapeutic strategy for cancer.

Since both organelles, mitochondria and lysosomes, share the power of majorly impacting the process of tumorigenesis, we will next further describe the main crosstalk between these two important organelles, shedding light on their interplay in cancer and their impact on cancer therapy.

## The Mitochondrial–Lysosomal Interplay in Cancer

In cancer, several important changes occur in all the organelles. However, the interplay between mitochondria and lysosomes is of high importance, because both organelles can interact to promote, in some cases cell death and in others tumorigenesis. There are two processes in which mitochondria and lysosomes work together: the first is LMP, a process in which enzymes from lysosomes can induce mitochondrial death pathway, and the second is mitophagy, a process in which lysosomes can degrade mitochondria, resulting in cell survival or cell death. Other than these two processes, there are several common effectors that play important roles in both organelles, which are also affected in cancer.

### Mitochondrial–Lysosomal Mechanisms

#### Lysosomal Membrane Permeabilization

As mentioned above, LMP is a mechanism that induces two types of cell death: apoptosis induced by partial and selective LMP, and necrosis provoked by the complete disruption of lysosomes. One of the causes of apoptosis during LMP is the activation of caspases in the mitochondrial death pathway by mitochondrial outer membrane permeabilization (MOMP) (65).

Reactive oxygen species (ROS) and cathepsins are well-known mediators of LMP-triggered cell death (81). It has been reported that only cathepsin D in the cytoplasm is enough to induce MOMP and apoptosis in human fibroblasts. However, cathepsin D alone is not in all cases of LMP sufficient to induce cell death (65).

**Figure 1:**
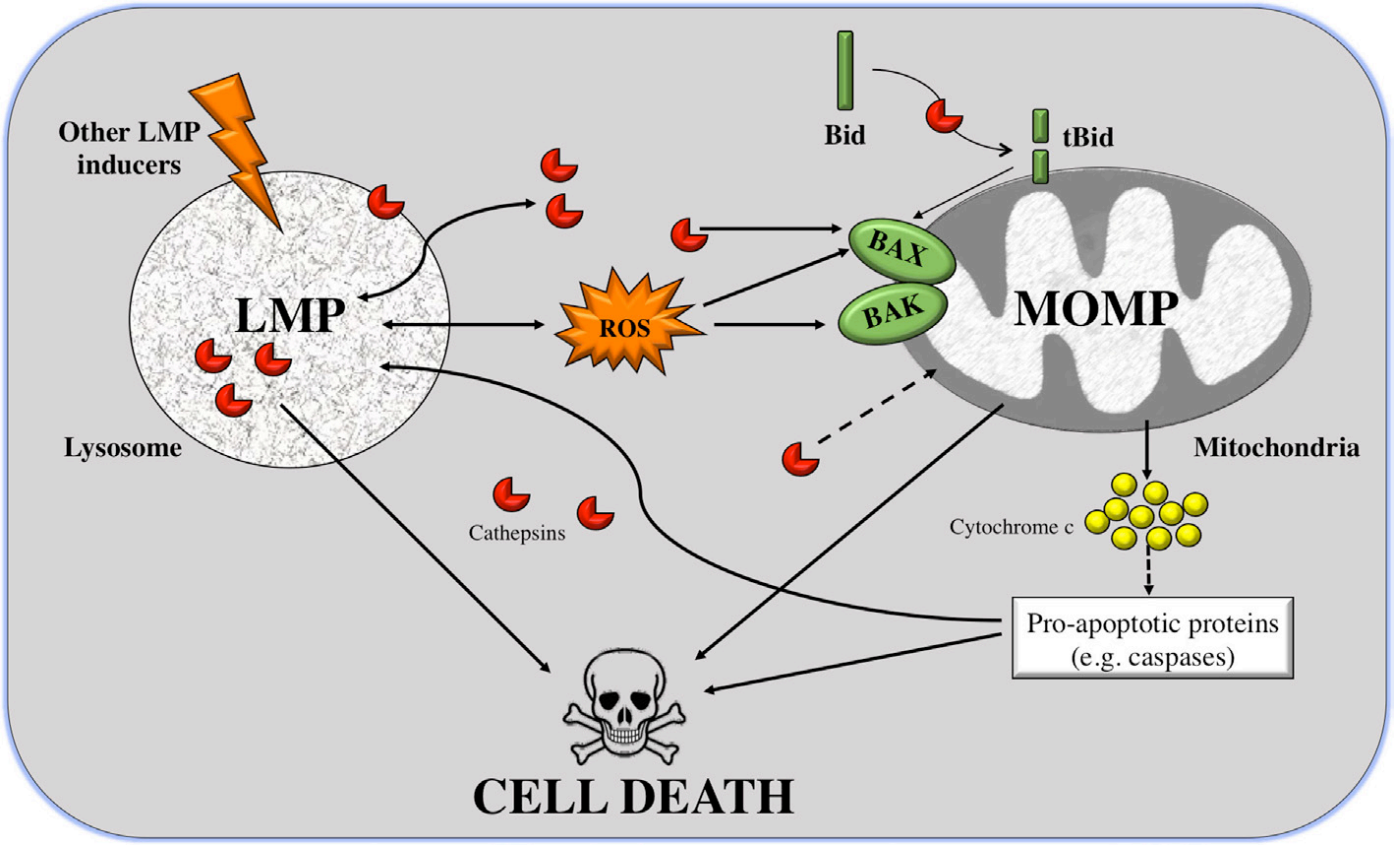
From lysosomal membrane permeabilization (LMP) to cell death through mitochondrial outer membrane permeabilization (MOMP). LMP is a process in which intralysosomal content (mostly cathepsins but also ROS) is leaked to the cytoplasm. Massive disruption of lysosomes induces cell death by necrosis, but selective LMP can induce apoptosis by MOMP. Cathepsins can promote, on the one hand, the cleavage of the pro-apoptotic protein Bid; and on the other hand, the translocation of Bcl-2-associated killer (BAK) and BAX to the outer mitochondrial membrane (OMM) where they form pores. Truncated Bid (tBid) can itself form pores to the OMM but can also activate BAK/BAX. These two processes can induce apoptosis, *via* release of cytocrome *c* and in a caspase-dependent way or independently of caspases. A positive feedback loop exists given that caspases and cathepsins are also inducers of LMP.

As shown in Figure 1, MOMP can be triggered by LMP in two manners: either Bid dependent or Bid independent. Bid is known as BH3-interacting-domain death agonist and belongs to the pro-apoptotic BH3-only Bcl-2 family. The Bid-dependent process occurs when Bid is cleaved by the active cathepsins at cytosolic pH (specially cathepsins B and D), after which Bid is capable to form pores at the OMM inducing MOMP and releasing cytochrome *c* from the mitochondria (81). Despite tBid is also known to activate BAX and BAK, in some cases, LMP can induce MOMP in a Bid-independent manner; cathepsins or stress stimuli can directly activate the proteins BAX and/or BAK (82, 83). After their activation and translocation to the mitochondria, BAX and BAK make pores at the OMM. This permits the translocation of numerous molecules bigger than 100 kDa, without inducing membrane rupture leading to apoptosis (84–86). Furthermore, cathepsin B has a major role in linking LMP to MOMP *via* the generation of lipid mediators, such as arachidonic acid that induces MOMP (87). Alternatively, other than ROS and cathepsins, there is a large list of agents capable of inducing this mitochondrial membrane permeabilization, such as sphingolipids, phospholipase A2, etc. (64). Some of these stimuli, like the pro-apoptotic proteins or caspases, are derived from mitochondria, suggesting that there is a positive feedback loop: mitochondrial damage also induces LMP (Figure 1).

In addition, the mechanism by which cathepsins are released from the lysosomes is not yet clear. There are three possible hypotheses: (i) through the rupture of the lysosomal membrane, (ii) through specific pores, or (iii) by special transporters. In an attempt to find which of the three hypotheses is valid, fluorescently labeled dextran molecules of different sizes were used. When inducing LMP, it was shown that only small molecules (size of 10 kDa) were released to the cytoplasm in most of the cells. In almost half of the cells, 40-kDa molecules were redistributed to the cytoplasm, and molecules larger that 70 kDa remained inside lysosomes. Based on the fact that cathepsins are relatively small proteins, around 40 kDa in size, it is inferred that cathepsins are among these released molecules. Furthermore, the low intralysosomal pH was maintained, suggesting that lysosomes were still active (88). However, it is still not enough to rule out any of the possible mechanisms.

Until now, several explanations may account for the higher vulnerability to LMP of cancer cell lysosomes. Since lysosomes are relatively large in cancer cells (89), one possibility would be that they are more prone to inducing cell death than lysosomes with normal sizes (90). Another possibility lies in the observation that cancer cells have higher metabolic rates. This is accompanied by an elevated turnover of proteins that contains iron, leading to iron accumulation in the lysosomes. Subsequently, these lysosomes will undergo an iron-mediated predisposition to a ROS-induced LMP (91). In other words, a characteristic of cancer cells is the increased levels of ROS, which is associated with an amplified release of cathepsins from the lysosomes. Since cancer cells appear to be more susceptible to LMP, its induction will eventually facilitate cancer cell death (92).

#### Autophagy and Mitophagy

Macroautophagy is a process in which intracellular proteins or organelles are degraded in the lysosomes. Degraded products are then released from lysosomes and recycled into biosynthetic and metabolic pathways. Through the elimination of those damaged components, autophagy basically provides quality control over proteins and organelles, as well as sustains mitochondrial metabolic function and energy homeostasis (93). More than 30 proteins coordinate the autophagic processes, generating autophagosomes from essentially all membrane sources from the cell. Autophagy-related genes (Atg) control the processes of autophagy. The products of Atg genes are regulated by nutrients (mTOR), energy [AMP-activated protein kinase (AMPK)], and stress [hypoxia-inducible factor (HIF)], which can turn the pathway on and off (94). Nevertheless, autophagy may also induce cell death, known as autophagic cell death (ACD). This specifically occurs when chromatin condensation is absent (95).

Autophagy’s role in cancer is still not clear. Some cancers are dependent on autophagy for survival and other cancers use autophagy as a mechanism of cell death. In some models, autophagy suppresses cancer initiation by evading the toxic accumulation of damaged organelles, specifically mitochondria. On the short run, this helps in limiting oxidative stress. On the long run, it restricts chronic tissue damage and oncogenic signaling. So, in this context, autophagy stimulation might help suppress and/or prevent cancer initiation. Though, other cancers depend on autophagy for survival. In order to fit the high metabolic needs of growth and proliferation, cancers (such as the pancreatic) use autophagy-mediated recycling to their own advantage (96). Hence, inhibiting autophagy in this case could be an insight for selective cancer therapy, since these tumors are more dependent on autophagy than normal tissues (93).

Degradation of entire organelles can also occur: mitophagy (mitochondria), reticulophagy (endoplasmic reticulum), lipophagy (lipid droplets), peroxophagy (peroxisomes), and xenophagy (microbes). Mitophagy or autophagy of mitochondria is required to eliminate dysfunctional mitochondria to maintain appropriate metabolic and cell survival signals (97). Here, we will focus only on mitophagy, a key process for the control of mitochondrial quality. It is of substantial importance for the normal development of cells and tissues. The most studied mechanism of mitophagy initiation involves the E3 ubiquitin ligase Parkin and the serine–threonine kinase PINK1 (PTEN-induced putative kinase 1). PINK1 is a mitophagy receptor found at the OMM that accumulates when mitochondria are damaged or undergo any stress leading to mitochondrial membrane potential loss. This PINK1 accumulation at the OMM recruits Parkin from the cytosol. Parkin ubiquitinates proteins at the OMM. These ubiquitinated proteins are recognized by p62, also known as Sequestrome 1 (SQSTM 1). P-62 binds to LC3/Atg8 and takes p62-containing aggregates to the autophagosome to be degraded (98).

In the recent years, the role of mitophagy in cancer has been extensively reviewed. Parkin is frequently genetically inactivated in cancer. Although certain cancers, such as sarcomas and uterine cancer, have amplifications in PARK2 gene, the majority of tumors with lesions in PARK2, including ovarian, breast, and lung cancers, harbor deletions or loss of function mutations. This is mainly because the PARK2 gene is found on a fragile location on chromosome 6 (99). Parkin has been evidenced to control cell cycle regulators, such as cyclin-dependent kinases (CDKs) and cyclins, promoting acceleration of cell cycle progression. It can also lead to the accumulation of damaged mitochondria and elevated ROS production, triggering DNA damage and tumorigenesis (100). In addition, PINK1 and Parkin can promote apoptosis through targeting and ubiquitinating anti-apoptotic Mcl-1 leading to degradation; they operate as molecular switches by dictating cell fate as a response to diverse cellular stresses (101, 102). However, mitophagy can happen in cancer cells without active Parkin, known as mitophagy-independent Parkin function. OMM proteins, such as FUNDC1, BNIP3, and NIX, are autophagy receptors, independently of ubiquitinization. Furthermore, other factors such as the phospholipid cardiolipin can induce mitophagy, as well as ubiquitin ligases, such as SMURF1 and MAPL (103–105).

During hypoxia, HIFs can induce mitophagy through the transcription of NIX and BNIP3. In addition to its transcriptional activation by HIF-1, FoxO3A, PPARα, RB/E2Fs, NF-κB, oncogenic Ras, and p53 also transcriptionally regulate BNIP3 (103), while NIX is transcriptionally regulated by HIF-1 and p53 (106, 107). Upon hypoxia or high oxidative phosphorylation, the small GTPase Rheb translocates to the OMM, where it can interact with BNIP3 and NIX to induce mitophagy, resulting in mTOR inactivation (108, 109). The role of BNIP3-dependent mitophagy in cancer presents some controversies. BNIP3-dependent mitophagy is required to limit mitochondrial mass and ROS levels in growing tumors; its loss leads to HIF-1α-dependent increases in tumor growth and increased progression to metastasis (110). However, other studies show that BNIP3 has a pro-tumorigenic role; its inactivation reduced cell migration and its upregulation suppressed the mTOR/S6K1 pathway. It is hypothesized that the dual role of BNIP3 can be explained by alternative splicing or variable transcriptional regulation *via* transcription factor Sp3 (111, 112).

Contrary to BNIP3, the role of NIX and FUNDC1 in tumor progression remains relatively unknown, requiring further investigation. They can induce mitophagy under hypoxic conditions (113). However, their role in cancer mitophagy is still to be raveled (107).

### Signaling Pathways Involved in the Mitochondria–Lysosomal Crosstalk

**Figure 2:**
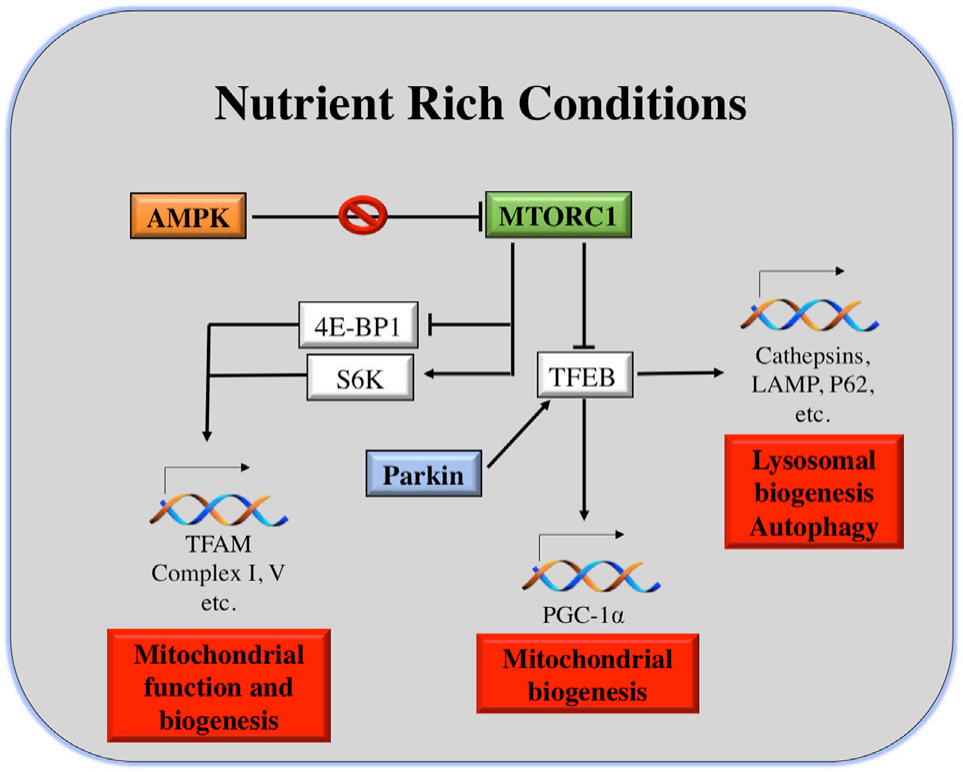
Mitochondria and lysosomes downstream mammalian target of rapamycin complex I (mTORC1). Under nutrient rich conditions, active mTORC1 can induce the transcription of different genes involved in mitochondrial function and biogenesis. At the same time, it can repress TFEB transcription factor, which is the main responsible for transcription of genes involved in lysosomal and mitochondrial biogenesis, autophagy, etc.

As shown in Figure 2, the master regulator of cell growth and metabolism mTORC1 and proteins of the same pathway are the main linkers of mitochondria and lysosomes. As described earlier, under nutrient-rich conditions, mTORC1 is activated at the lysosomal surface. mRNA translation of many genes occurs, *via* the activation and repression of S6K and 4E-BP1, respectively. Among the genes repressed by 4E-BP are TFAM, Complex I and Complex V of the mitochondria, regulating mitochondrial activity and biogenesis (114).

On the other hand, under starvation conditions, mTORC1 is inhibited and TFEB positively regulates the expression of lysosomal and autophagy genes, as well as the expression of PGC-1α. PGC-1α coactivates numerous biological programs in diverse tissues; it is a key regulator of lipid metabolism (115), but also it promotes mitochondrial biogenesis (30). Notably, TFEB activation can be induced by mitophagy as well, in an attempt to induce mitochondrial biogenesis after eliminating malfunctioning or damaged ones. The mechanism of TFEB activation by mitophagy is different than that under starvation conditions. Parkin promotes TFEB nuclear translocation, inducing lysosomal and mitochondrial biogenesis (98). In addition to that, upon energy depletion, AMPK is activated. AMPK serves as a fuel gage as it becomes active when ATP/AMP ratio is low, thus maintaining energy homeostasis (116). AMPK inhibits mTORC1 by the direct phosphorylation of Raptor, a molecule of the mTORC1 complex. This explains why AMPK has been considered as a tumor suppressor. AMPK activation is essential for increased mitochondrial biogenesis under glucose-limited conditions, since AMPK activation increases the expression of PGC-1α and TFAM (117) (Figure 2).

When mTORC1 is active, there is an increase of mitochondrial biogenesis. This increase in mitochondrial biogenesis will result in a gain of ATP production capacity, a mandatory energy source for translation (114). By contrast, mitochondrial biogenesis induced by AMPK is an attempt to accelerate ATP generation, for restoring its level in favor of cell survival. This takes place under limiting nutrient availability or metabolic stress (118).

To further emphasize the link between mitochondria and lysosomes, it has been shown that mTOR not only binds to lysosomes (when mTORC1 is activated when amino acids are present) but it can also be associated with MOM. This is needed to integrate different stress signals that affect the function of mitochondria and regulate a checkpoint implicating p70S6K, one of the well-known targets of mTORC1 (119). Recent studies link mitochondrial dynamics to the equilibrium between nutrient supply and energy demand, suggesting variations in mitochondrial architecture as an adaptive mechanism to metabolic demands (120).

Another important regulator is Rab7, which belongs to the RAB family, a RAS-related group of GTP-binding proteins. This family of proteins includes important regulators of vesicle transportation and is localized in certain intracellular compartments (121). Numerous studies indicate that Rab7 plays major roles in controlling maturation of endosomes and transportation to lysosomes, as well as in phagocytosis, retromere regulation, cytoskeleton regulation, autophagy, and mitophagy (122, 123).

Normally, Rab7 is found on late endosomes and this acquisition is complemented by Rab5 loss, an early endosome marker. The switch from Rab5 to Rab7 is a process in which both proteins cooperate sequentially and dynamically. This determines Rab5 recruitment to early endosomes and Rab7 recruitment and Rab5 loss at late endosomes (124–126). Late endosomes can fuse with lysosomes and other late endosomes, only if the Rab5 to Rab7 switch is accompanied by variations in fusion and tethering machinery. This allows direct contact in between organelles (127).

**Figure 3:**
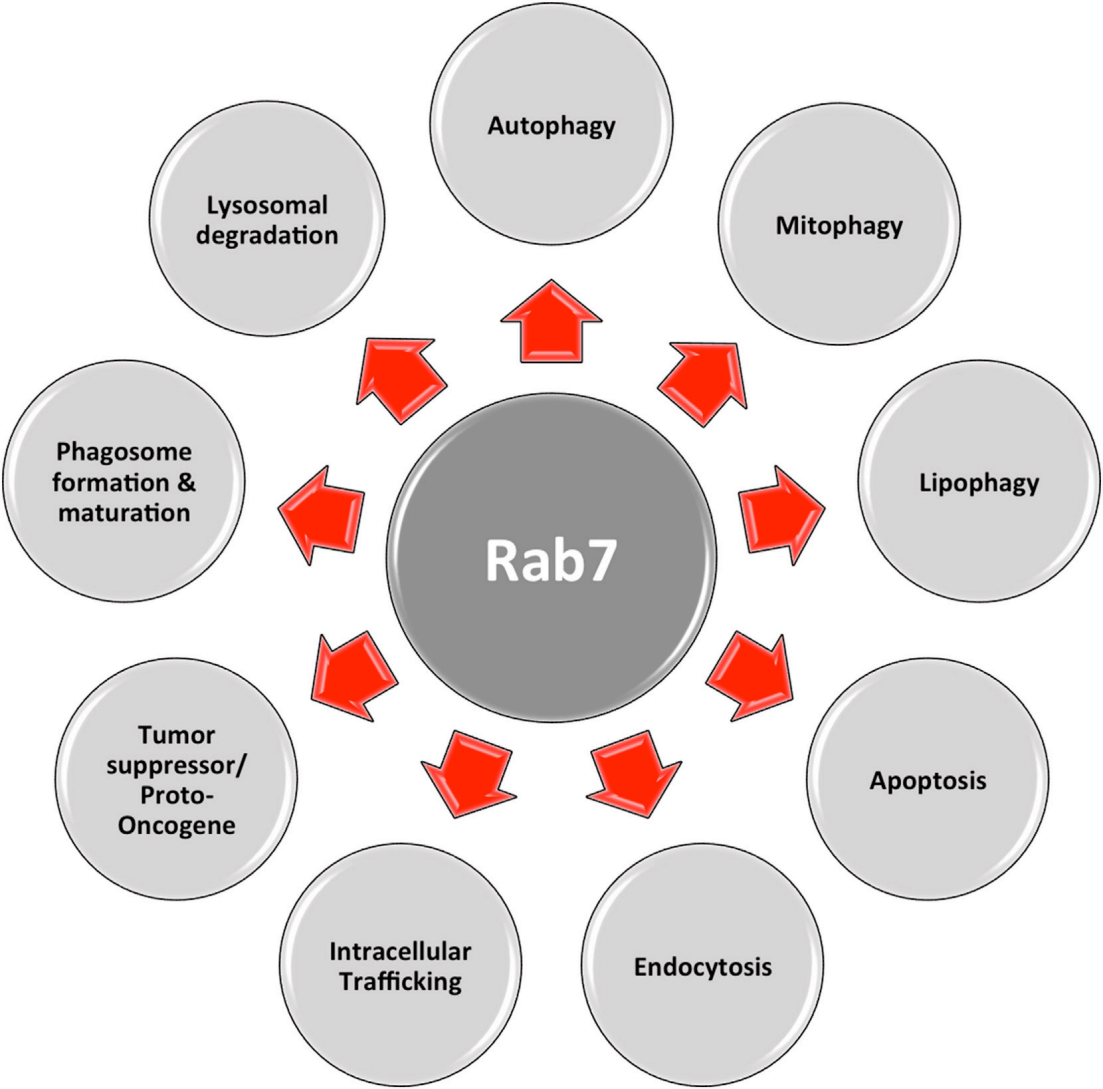
Multifaceted Rab7. Rab7 has distinct roles in numerous vital cellular functions: phagosome formation and maturation, lysosomal degradation, autophagy, mitophagy, apoptosis, etc.

Other than being a marker of late endosomes, Rab7 is vital for mitophagy (Figure 3), as it is a downstream effector of Parkin (128). This occurs with the help of TBC1D15/17 and Fis1. The first protein belongs to the TBC family (Tre2/Bub2/Cdc16), having RabGAP functions (129, 130), while the latter is a fission protein with cytosolic N-terminal, bound to the OMM at its C-terminal (131, 132). In the absence or inactivity of TBC1D15, membranes that are labeled with LC3 excessively accumulate and lose their cargo orientation. In turn, membrane tubules are sent along microtubule tracks away from the mitochondria. Therefore, it is inferred that, during mitophagy, TBC1D15 binds to Fis1 and LC3 inducing Rab7 activity, which leads to the shaping of the autophagosome isolation membrane. In fact, besides promoting microtubule-associated trafficking and autophagosomal membrane growth, the activity of Rab7 is affected by TBC1D15/17 activity, inducing autophagosomal membrane expansion to correctly surround the mitochondria (128). This indicates that, in case of Parkin-regulated mitophagy, Rab7 is essential for expanding LC3-labeled isolation membranes. Otherwise, inactive Rab7 might help in mediating the release of LC3-positive membranes from microtubules (133). The above-described model substantially differs from the well-known role of Rab7 in controlling autophagosome maturation and fusion with lysosomes (134, 135). In addition, it was shown an increase in the interaction between Rab7 and Mitofusin2 (MFN2), a mitochondrial fusion-related protein, as a response to starvation. This suggests the contribution of Rab7 during autophagosomal membrane maturation, as an adaptor protein used by MFN2 (136). Hence, Rab7 has a dual role in mitophagy, i.e., autophagosome formation and maturation.

As shown in Figure 3, this multifaceted small GTPase also participates in other important processes in the cell, such as in apoptosis and the activation of stress response pathways (137). One of these pathways is the mTORC1 pathway, through which mTORC1 moves toward a Rab7-containing compartment in the presence of amino acids (138). The direct interaction between Rab7 and mTOR has been proved by co-immunoprecipitation experiments (139). During bioenergetic stress, several groups showed a Rab7-dependent lysosomal crosstalk with apoptosis and its regulatory machinery, i.e., intramitochondrial recruitment of endolysosomes mediates apoptosis. As explained before, in the presence of growth factors, mTOR and AKT are activated initiating downstream signaling cascades. Under these conditions, nutrient transporter proteins facilitate the import of extracellular nutrients, supporting cellular bioenergetics by supplying the mitochondria with metabolic substrates. In this case, one important apoptotic mediator, cytochrome *c* is retained at the mitochondrial intermembrane. When there is nutrient starvation, these same signaling cascades are silenced, and genes are no longer transcribed. Existing transporters are trafficked to the lysosomes by Rab7, where they are degraded and removed from the cell. This decrease in cellular bioenergetics results in substrate limitation at the mitochondrial site, loss of homeostasis, and cytochrome *c* release, eventually leading to apoptosis. This process can be rescued by inhibiting Rab7. For example, transporter proteins destined to enter the endocytic pathway and be trafficked to lysosomes for degradation, instead, are recycled and re-expressed on the cellular surface. As a result, extracellular nutrients are continually imported, and cellular bioenergetics is maintained, as well as mitochondrial homeostasis, in the absence of growth factors (140). In this case, Rab7 has a proapoptotic function, by limiting cell autonomous uptake of extracellular nutrients (141).

In cancer, the specific role of Rab7 is not fully understood. In the literature, Rab7 has been depicted as either a tumor suppressor (68, 141, 142) or a proto-oncogene (143–146), depending on tumor type, morphology, and metastatic and invasive abilities (122) (Figure 3). Particularly, synergy between HSP90 inhibition and Rab7 depletion decreases EGFR and Her2 levels, through proteasomal degradation, and promotes apoptosis, depicting a proto-oncogenic role of Rab7 (147, 148). During melanoma development, Myc is activated, inducing Rab7 overexpression. Subsequently, Rab7 expression is downregulated to support the invasive and metastatic characteristics of melanoma (145). Moreover, the knockdown of Rab7 in prostate cancer cells led to the overexpression of c-Met, a protein involved in the promotion of cell invasion and metastasis (149).

In fact, since Rab7 is mainly accountable for intracellular trafficking, which is linked to the metastatic/invasive ability of tumors, and the degradation of many organelles and molecules, such as adhesion molecules and signaling receptors, it is a key regulator in governing cellular homeostasis. Certainly, Rab7 is a central molecule of cell survival, differentiation, and apoptosis. Current data suggest that the regulation of Rab7 expression and activity can reduce several pathologies, such as cancer (122). Of course, further work will be needed to investigate this possibility.

## Therapeutic Approaches in Cancer Treatment

Cancer cells exhibit a significant number of metabolic alterations associated with mitochondria, lysosomes, and other sub-cellular organelles. These organelles exhibit a number of deregulations, which have been identified as potential drug targets for successful rational drug design and therapy. For their involvement in bioenergetics, redox balancing, and survival, targeting mitochondria for therapeutic benefits is already in practice to induce apoptotic cell death (150, 151). In addition, the advances in lysosome research have highlighted their importance for degradation, signaling pathways, and cell death in pathophysiological conditions; thus, targeting lysosomes has also been considered a new therapeutic strategy for cancer treatment (76).

In this review, we highlighted the interplay between lysosomes and mitochondria and its importance in cell fate. We propose that targeting this crosstalk between both organelles might be crucial for fighting cancer.

### Inducing LMP

Indeed, many compounds are described to induce LMP and subsequent cell death in various human cancer cells and animal models but are not in clinical use (152, 153). Besides, little is known about the endogenous inhibitors preventing LMP and which mechanisms suppress lysosomal hydrolases in the cytoplasm of both, normal and cancer cells. Inducing LMP-dependent death could activate self-destructive processes in tumor cells, particularly if those cells were dependent on such inhibitors. Future investigations are needed to clarify if antagonists of LMP inducers may be useful synergistically with the current clinical treatments (64).

Interestingly, a minimally invasive anticancer modality called Photodynamic therapy (PDT) is able to induce LMP. PDT combines a drug (a photosensitizing agent) with a precise light wavelength, inducing ROS generation and killing tumor cells (154). The location where the photosensitizing agent is directed is very important, as it determines where the primary damage occurs. Usually photosensitizing agents accumulate either in mitochondria, inducing rapid apoptosis, or in lysosomes, inducing LMP and subsequent cell death (64). To date, PDT is used for treating or relieving the symptoms of non-small cell lung cancer patients and esophageal cancer patients. PDT still presents some limitations, since only tumors on the skin or just underneath it, or in internal organ linings and/or cavities can be treated with this technique. But it cannot be used for treating large tumors or metastasis (155–158).

### Targeting Mitophagy

Targeting mitophagy as an approach to adjuvant chemotherapy has been already questioned by Chourasia et al. (107). They claim that the deletion or inhibition of Parkin and BNIP3 induces the Warburg effect, thus favoring tumorigenesis. Nevertheless, acute chemical inhibition of mitophagy is still an effective approach for advanced tumors that have switched to glycolytic metabolism but still depend on mitochondria for further metabolic purposes (107). Of course, this approach has still to be therapeutically tested.

### Targeting Common Effectors between Mitochondria and Lysosomes

Other than targeting processes in which mitochondria and lysosomes are linked, inhibitors of their main common effectors, i.e., AMPK or mTOR have been already tested for cancer therapy. The role of AMPK in cancer cells is paradoxical. It can be a tumor suppressor, but can also promote tumorigenesis, stimulating cell survival in glucose-deficient situations and preserving metabolic homeostasis (117). Despite that, the use of the anti-diabetic drug Metformin, non-steroidal anti-inflammatory drugs, such as Aspirin, AICAR, and some natural products known to be AMPK activators, has shown to decrease tumorigenesis in animal models and cancer cell lines (159, 160). In addition, preclinical evidence suggests that Metformin appears to prevent the proliferation and growth of certain tumor types. There are currently more than 100 ongoing or clinical studies assessing the role of metformin in the therapy cancer (161, 162). It is well understood that metformin targets the mitochondrial complex I. However, it has been suggested that metformin could directly influence the V-ATPase activity of lysosomes (163), so this fact further supports the importance of lysosomal–mitochondrial link for cancer treatment.

Targeting mTOR may be crucial for cancer treatment not only for cell growth and proliferation but also for reversing the Warburg effect characteristic of tumor cells (164). At the molecular level, mTORC1 inhibition may induce mitochondrial biogenesis *via* PGC-1α, as well as repression of transcription of mitochondrial genes *via* 4E-BP1 (114) depending on the model. mTORC1 inhibition induces lysosomal biogenesis and also initiates several feedback loops to upstream pathways, activation of which might be beneficial for the survival of tumor cells and metastasis. The best-known mTOR inhibitor is Rapamycin, which does not inhibit directly mTOR’s kinase (catalytic) activity. Together with FKBP12, it binds specifically to mTORC1 (at high concentrations also to mTORC2). The binding occurs next to the kinase active site. Consequently, it can only inhibit a number of mTORC1 functions. Given this, and the importance of mTOR for cancer, several groups have developed other inhibitors to target mTOR’s catalytic subunit (PP242, Torin 1 and 2, etc.). As reviewed by Xie and al., some mTOR inhibitors are already in clinical trials for treating cancer (165). Despite that, the utility of such inhibitors in oncology still appears to be limited, given that autophagy can be induced by mTOR inhibition, thus promoting cancer cell survival.

As reviewed above, RAB7 is a prominent target for cancer treatment (143). In addition, there are already drugs that target RAB7. It has been shown that liensinine, a major isoquinoline alkaloid that inhibits RAB7A recruitment to lysosomes, not autophagosomes. In this way, autophagy/mitophagy is impaired, enhancing the efficacy of chemotherapy in breast cancer cell lines (166).

### Using Nanomedicine for Inducing Cell Death

Nanotechnology is the science of controlling matter, at the molecular level, to generate devices with new biological, physical, and/or chemical characteristics. It is in the spotlight of therapeutic innovation. The use of nanomaterial is a particularly promising tool not only to improve the diagnosis but also to generate new cancer treatments and overcome the drawbacks of traditional therapies (167). In particular, the discovery of gold nanorods (GNRs) has provided a new method to induce apoptosis specifically in cancer cells, while posing a negligible impact on normal cells. They are able to induce apoptosis in cancer cells through lysosomal permeability, as indicated by cathepsin D release, and a decrease in mitochondrial membrane potential (168). These findings are promising for the further implementation of nanotechnology at the clinical practice.

## Conclusion and Future Perspectives

Intracellular organelles, as thoroughly discussed, are the major players of cellular networks. Even though physical contact among these organelles was exhaustively described through out the years, research is now shifting toward revealing the crosstalk of these entities on the signaling levels, as well as their physiological relevance. Mainly, organelle interactions are needed for metabolite exchange, and more interestingly, in membrane dynamics, intracellular organelle distribution, and the assembly of dynamic signaling platforms depending on cellular requirements.

In tumors, cells significantly display metabolic aberrations, associated directly or indirectly with mitochondria and lysosomes. These anomalies promote cancer cell growth and survival, while exhibiting distinctive properties that render cancer cells vulnerable to specific anticancer agents. In other words, the deregulation of these organelles in cancer cells as compared to their counterparts in healthy cells is a main reason for promising targeted drug therapy. Though substantial advancement has been made regarding elucidating the role of these anomalies in oncogenesis and chemotherapy-resistance, a better interpretation of the main pathophysiological differences between organelles of normal and tumor cells can undoubtedly compliment the efforts to improve selective targeted anti-cancer agents.

Lysosomes and mitochondria have common regulators and can physically interact to maintain cell homeostasis or induce cell death. However, in cancer everything becomes a paradox; all of the processes in which lysosomes and mitochondria interact, except for LMP, and the common regulators (mTOR, AMPK, Rab7, etc.) present a dual role: on the one hand, they can promote tumorigenesis and, on the other hand, they can induce cell death. The relative contribution of these pathways would depend on tumor type, state, metastatic ability, microenvironment, metabolic reprograming, etc. This reflects the importance of these two organelles for cancer treatment. New potential targets have been proposed, i.e., PGC-1α, TFEB, Rab7, etc. However, will these targets overpass the problem of having a paradoxical role in cancer treatment? As we have seen already, every case needs to be studied independently, in order to predict whether the treatment would be beneficial or not. This is known as personalized medicine.

## Author Contributions

LC, AN, and LC discussed the ideas and wrote the paper.

## Conflict of Interest Statement

The authors declare that the research was conducted in the absence of any commercial or financial relationships that could be construed as a potential conflict of interest.
